# Ionic Liquids in Tribology

**DOI:** 10.3390/molecules14062286

**Published:** 2009-06-24

**Authors:** Ichiro Minami

**Affiliations:** Department of Chemical Engineering, Iwate University, 4-3-5 Ueda, Morioka, Iwate, 020-8551, Japan; E-Mail: ichiro@iwate-u.ac.jp

**Keywords:** synthetic lubricant, surface chemistry, molecular design, friction and wear, thermo-oxidative stability

## Abstract

Current research on room-temperature ionic liquids as lubricants is described. Ionic liquids possess excellent properties such as non-volatility, non-flammability, and thermo-oxidative stability. The potential use of ionic liquids as lubricants was first proposed in 2001 and approximately 70 articles pertaining to fundamental research on ionic liquids have been published through May 2009. A large majority of the cations examined in this area are derived from 1,3-dialkylimidazolium, with a higher alkyl group on the imidazolium cation being beneficial for good lubrication, while it reduces the thermo-oxidative stability. Hydrophobic anions provide both good lubricity and significant thermo-oxidative stability. The anions decompose through a tribochemical reaction to generate metal fluoride on the rubbed surface. Additive technology to improve lubricity is also explained. An introduction to tribology as an interdisciplinary field of lubrication is also provided.

## Introduction to Tribology

“Tribology” might be an unfamiliar term for the readers of this journal. It was coined after the Greek word “tribos” which means “rubbing”. Tribology is defined as the science and technology of interacting surfaces in relative motion and of associated subjects and practice [1]. Formerly, it was known as the study of friction and lubrication engineering. This new definition of tribology aims at establishing new engineering concepts by integrating individual technology and engineering related to friction. Therefore, tribology is an interdisciplinary field ranging from fundamental research to industrial applications. Nanotechnology and surface sciences are some examples of fundamental or academic research in tribology. Tribology in practice is of considerable importance. It has been estimated that the appropriate application of tribological principles and practices in industry can lead to savings of 1.0% to 1.4% of a country’s gross national product (GNP) [2]. Research in tribology is being conducted mainly from the viewpoint of mechanical engineering. Recently, the role of surface chemistry in tribology has attracted the attention of researchers, and the keyword “tribochemistry” is now frequently found in tribology journals.

We use various kinds of devices and machines in our daily lives. These devices and machines consist of several moving parts that need lubricants for smooth functioning. A reduction in the friction coefficient improves the energy efficiency of a device. Prevention of wear prolongs the lifetime of mechanical parts. Lubricants are classified into three categories, namely, lubricating oils (liquid lubricants), greases, and solid lubricants (self-lubricating materials). Lubricating oils are most commonly used in machines. When a viscous liquid is introduced between two friction surfaces, a liquid film is developed between the surfaces and consequently, hydrodynamic lubrication is achieved. The liquid film prevents direct contact between the friction surfaces, thereby reducing friction. In this lubrication mode, the viscosity of the lubricant, applied load to the contact, and sliding velocity are the major factors that decide the lubrication performance. Viscous liquids are good lubricants because they form a stable liquid film and thus prevent contact between solid surfaces. Therefore, viscosity is one of the important properties of lubricating oils. When the lubrication condition becomes severe, the liquid film breaks down and the friction surfaces directly come in contact with each other. This is defined as boundary lubrication. Friction and wear under boundary lubrication are considerably higher than those under hydrodynamic lubrication.

Direct contact between friction surfaces causes wear of the materials. This leads to the formation of an exposed chemically active nascent surface. Furthermore, significant friction heat is generated under boundary lubrication. These factors occasionally activate certain chemical reactions in a lubricant present between the rubbing surfaces. Such reactions are called tribochemical reactions or mechanochemical reactions. These reactions usually cause degradation of the lubricant. However, under certain conditions, they are known to form a protective film over the rubbing surfaces. In other words, tribochemical reactions can be beneficial and detrimental for lubrication. Presently, tribochemical reactions have not been understood clearly and many efforts are being made by tribochemists to understand the mechanism of these reactions.

In addition to reducing friction and wear, lubricating oils are required to cool the rubbing surfaces that are heated due to friction. Thermo-oxidative stability of lubricants is essential to minimize degradation during service and storage. Lubricating oils may vaporize and sometimes may catch fire due to friction heat. Therefore, less volatile and nonflammable liquids are desirable for lubricants. Another requirement for lubricating oils is compatibility with tribomaterials; lubricating oils should not cause corrosion of metals and swelling of polymers.

Lubricating oils have been used since ancient times. In ancient Egypt, it was empirically known that friction could be reduced when certain liquids were applied between surfaces in relative motion [3]. Natural oils such as vegetable oils were used as lubricating oils. In the middle of the 19th century, humans discovered petroleum, which proved to be a versatile source of energy and materials. Since then petroleum-based lubricants, also called mineral oils, have been developed. Presently, these lubricants are used for general purposes and are available at a reasonable cost. Besides mineral oils, synthetic fluids intended for use as high performance lubricants are also available. Examples of synthetic lubricants are polymeric/oligomeric alkenes, Fischer-Tropsch hydrocarbons, esters, and polyethers.

Recently, ionic liquids have received attention as novel functional fluids. Their advantages are well described in the rest of articles of this special issue. Nonvolatile and nonflammable liquids that possess lubricating properties are highly desired in tribology. This article reviews the fundamental attempts to use ionic liquids in lubrication engineering.

## Nomenclature

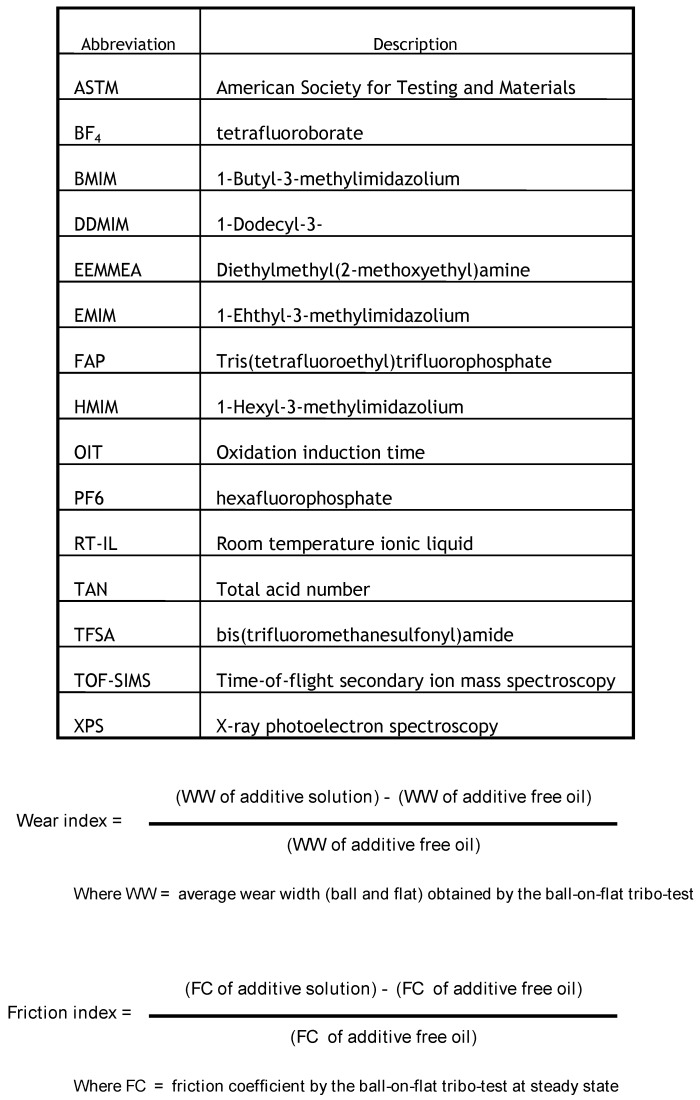



**Table 1:** Reported anions and cations mentioned in articles on tribology.

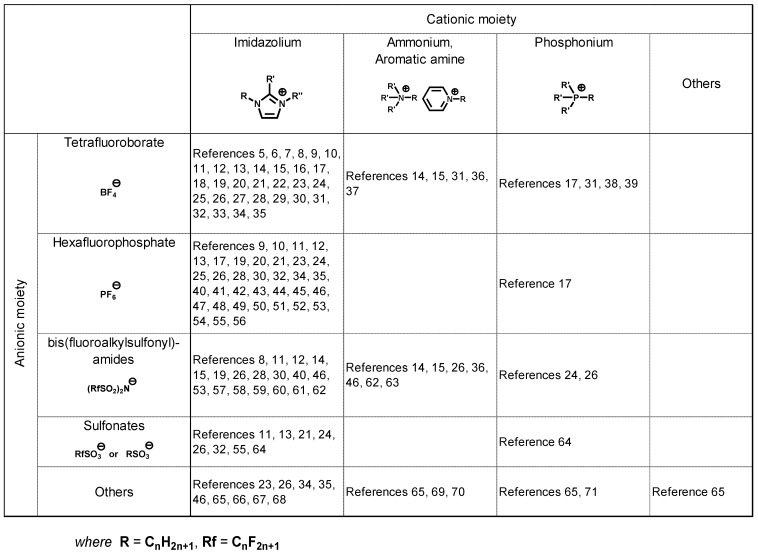

## Literature survey of ionic liquids as lubricants

An article on the use of molten salts (ionic liquids) as lubricants was first published in 1961 [4]. A mixture of LiF, BeF_2_, and UF_4_ in a molar ratio of 62:37:1 was melted at 460 °C. The mixed salt was subjected to a high-temperature bearing test at 650–815 °C. It took almost 40 years before room temperature ionic liquids (RT-ILs) were recognized as lubricating fluids [5]. Since then, approximately 70 articles on tribology of RT-ILs have been published in international journals. These articles have reported on the evaluation of RT-ILs by laboratory tribotest procedures. Most of the RT-ILs mentioned in these articles exhibit tribological properties comparable to those of conventional synthetic lubricants. The chemical structure of the anionic and cationic moieties described in these articles is summarized in Table 1. The major anions reported in these articles were trifluoroborate (BF_4_) and hexafluorophosphate (PF_6_). However, this does not imply that BF_4_ and PF_6_ are suitable anions for lubricants. These anions are easily available as reagents or industrial samples at a reasonable cost. Hydrophobic anions such as bis(trifluoromethanesulfonyl)amide (TFSA) exhibit better tribological properties compared to BF_4_ and PF_6_ for steel-steel contact. With regard to cationic moieties, imidazolium-derived moieties were present in the majority of examples. Various synthetic reactions that introduce alkyl groups and/or functional groups in the imidazole ring are well known. Therefore, imidazole is a versatile building block for designing molecules that have appropriate chemical and physical properties. The thermal stability of imidazole ring [72] is also beneficial for lubricants.

Steel is a material that is widely used in machines. Therefore, the tribological properties of ionic liquids were evaluated for a steel-steel contact. Lightweight materials such as aluminum alloys and silicon are difficult to lubricate. Lubrication of these materials by RT-ILs has been examined previously (Table 2).

**Table 2:** Reported tribo-material for ionic liquid lubrication.

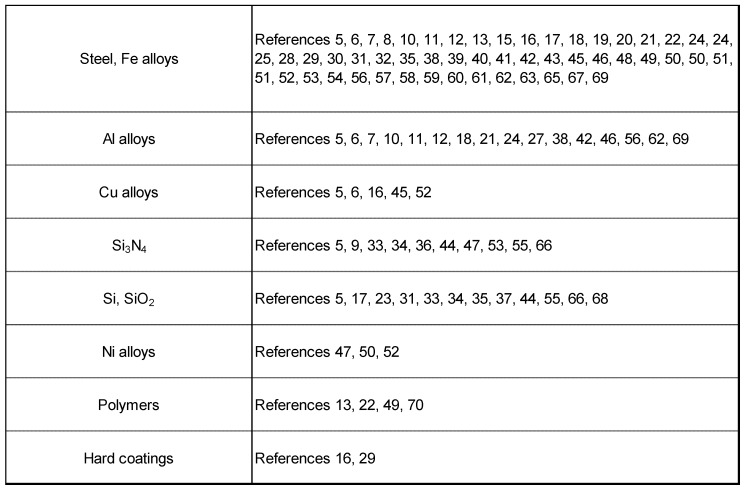

## Relationship between molecular structure and tribological properties of RT-ILs

**Figure 1:**
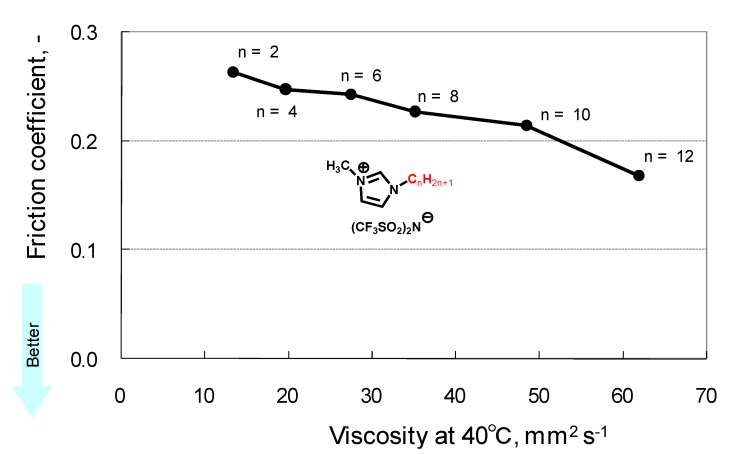
Effect of chain length in imidazolium cation on friction.

**Figure 2:**
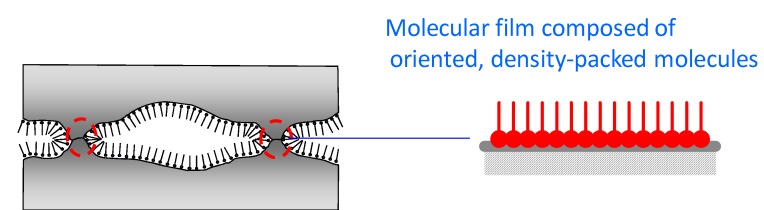
The Bowden-Tabor type boundary film model.

There are a number of standard procedures and specifications for evaluating lubricants [73]. However, they have been developed only for fully formulated lubricants (i.e. overall performance of the lubricant, including durability, was optimized by additive technology) such as engine oils. Although some of these procedures and specifications are applicable for fundamental research, most of them have stringent criteria, which make it impossible to evaluate the tribological properties of nonformulated base fluids. Until now, most researchers have evaluated RT-ILs using their own procedures. Therefore, the reported data cannot be compared on a single scale. Under these circumstances, structure-performance relationships of ionic liquids in tribology are highly desired as they are recognized as designer fluids. For example, 1-alkyl-3-methylimidazolium was selected as a model cation. TFSA salts of the cation were prepared, and their tribological properties were evaluated by a pendulum type friction test. As shown in Figure 1, the friction coefficient decreases with an increase in the chain length of the alkyl group [15,57]. One plausible explanation for this result is that the viscosity increases with the increase in chain length. In addition, a longer alkyl chain is beneficial for preventing direct steel-steel contact. This results in the reduction in friction between the two surfaces. This mechanism is also known as the Bowden-Tabor model in which density-packed adsorption of lubricant molecule on friction surfaces is essential [74]. The schematic representation of the model is shown in Figure 2.

**Figure 3:**
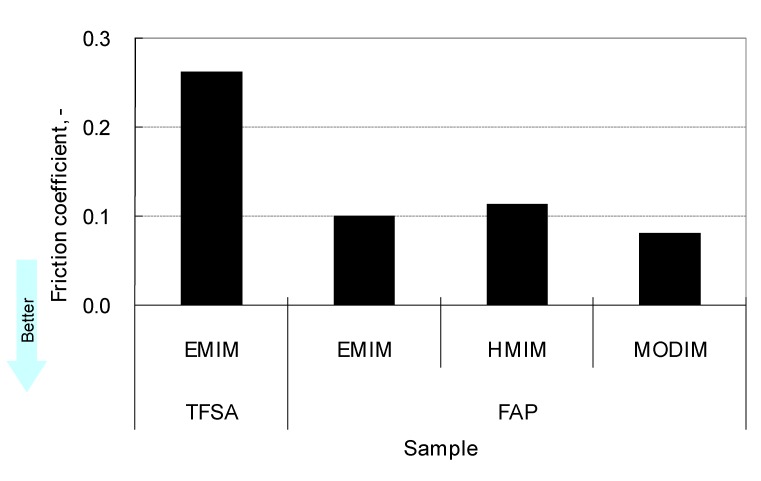
Effect of anion on friction.

**Figure 4:**
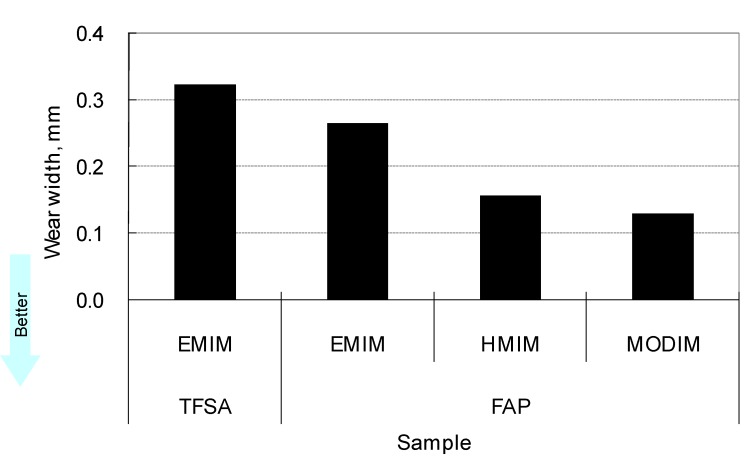
Effect of anion on wear.

The anions have a significant influence on the tribological properties of ionic liquids. Hydrophobic anions such as BF_4_ and PF_6_ occasionally cause corrosion of steel under humid conditions. PF_6_ decomposes to generate hydrogen fluoride by hydrolysis. In contrast, other hydrophobic anions are less corrosive and exhibit good tribological properties [64]. Tris(tetrafluoroethyl)trifluorophosphate (FAP) is more hydrophobic than TFSA (Table 3). Furthermore, friction and wear caused by FAP salts are considerably lower than those caused by TFSA salts (Figure 3, Figure 4) [65]. 

**Table 3:** Solubility of water in HMIM derived ionic liquid.

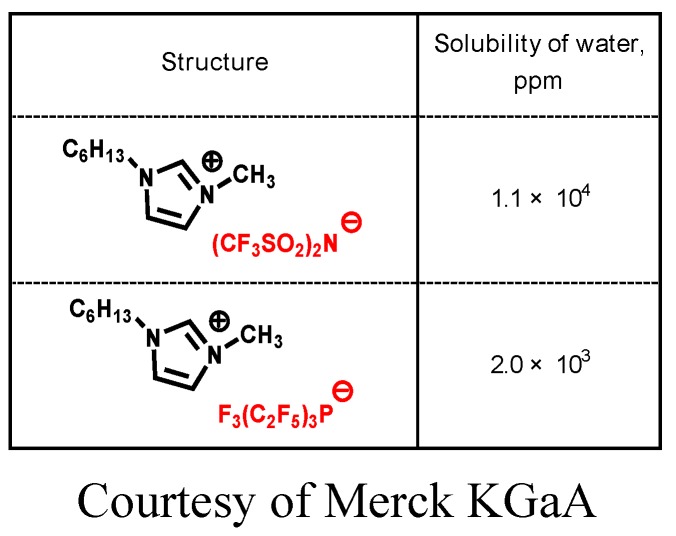

**Figure 5:**
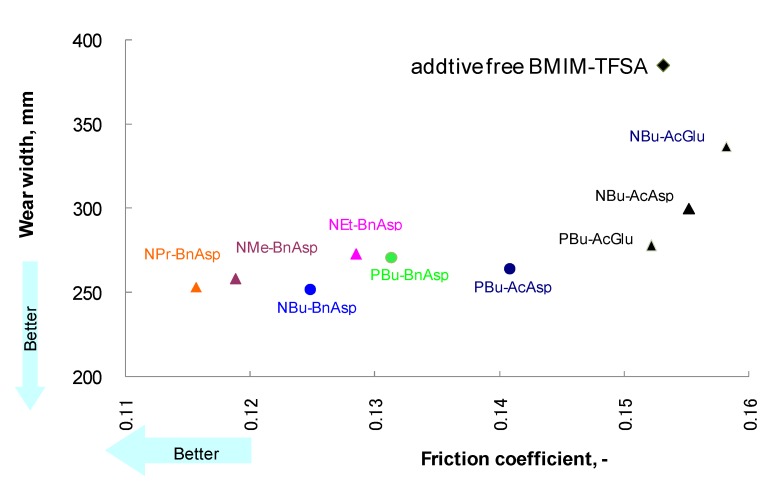
Additive effect on friction and wear for [BMIM]TFSA.

**Figure 6:**
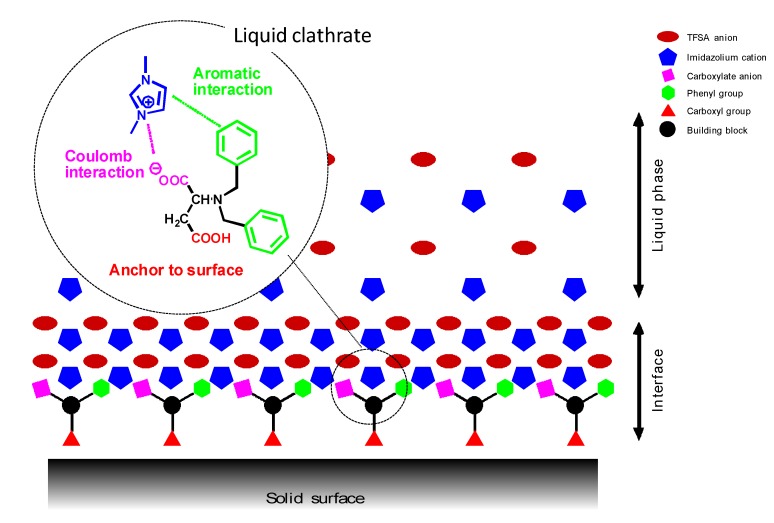
Proposed structure of boundary film provided by the additive and imidazolium-derived ionic liquids.

Usually lubricating oils are optimized by additive technology in order to meet the requirements for practical applications. Although various lubricant additives are available at a reasonable cost [75], most of them were developed for mineral oils, and hence they hardly dissolve in ionic liquids [40]. Simple compounds such as carboxylic acids [15,57] and benzotriazole [45,51] are considered as potential additives for ionic liquids.; however, their thermal stability and volatility make them unsuitable for addition to ionic liquids. Amino-acid-derived salts (Table 4) were developed as additives for ionic liquids [60]. As shown in Figure 5, the addition of aspartic acid derivatives to [BMIM]TFSA significantly reduces friction and wear by 20% and 40%, respectively. These additives were easily dissolved in imidazolium-derived ionic liquids through ionic interactions. The carboxyl group in the additive has an affinity toward steel surfaces. This functional group acts as an anchor for adsorption of molecules on the surface. The relationship between the molecular structure and tribological properties suggests the formation of a protective film comprising both additive and base oil molecules. *N*-benzyl-protected additives possess better anti-wear properties than *N*-acetyl-protected ones. It is possible that there is an interaction between the phenyl group and imidazolium ring. This would facilitate an interaction between the base oil and the surfaces through the additive molecules. The results of the tribotest support the proposed mechanism as shown in Figure 6 [61].

**Table 4:** Structure of amino acid derived additives for ionic liquids.

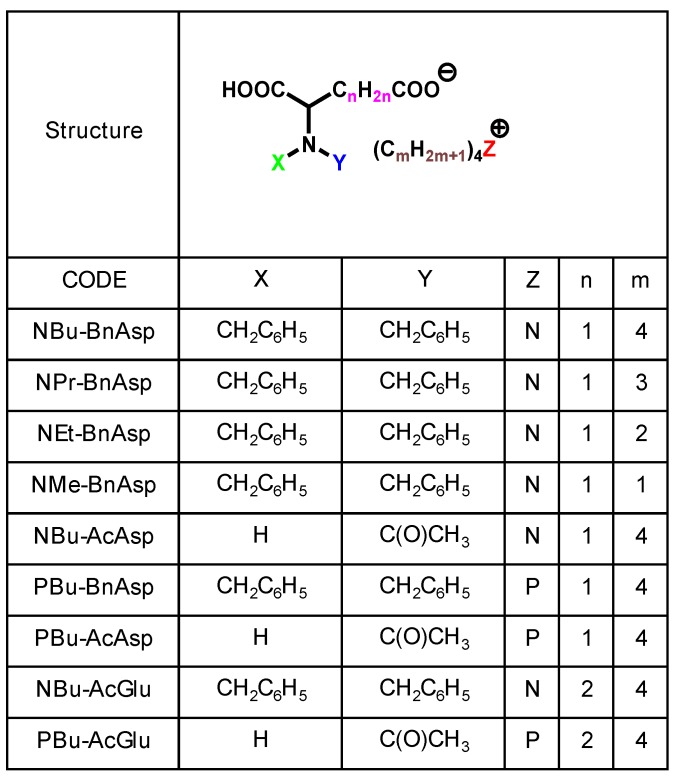

**Figure 7:**
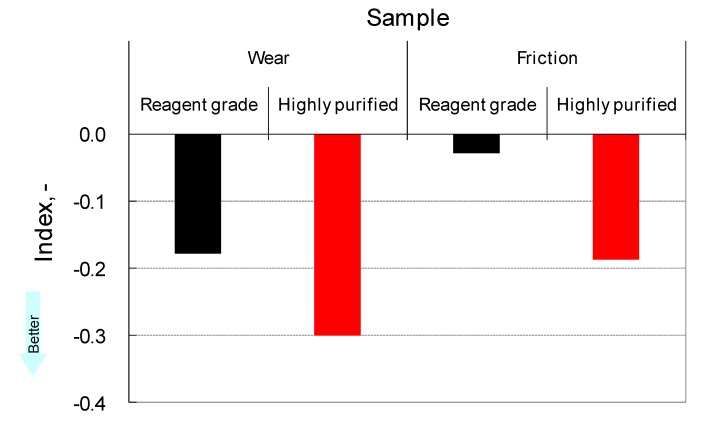
Influence of purity on additive response. (See nomenclature for “index”).

It should be noted that the purity of ionic liquids is of significant importance for improving the tribological properties by additive technology. As shown in Figure 7, highly purified [BMIM]TFSA and NBu-BnAsp provides significant lubricity, while reagent grade [BMIM]TFSA provides acceptable lubricity. However, no detectable impurities were found in reagent grade [BMIM]TFSA. This subject is still under investigation. From previous studies, the dependence of additive response on the refining grade of base oil is well known [76].

## Thermo-oxidative stability of ionic liquids

**Figure 8:**
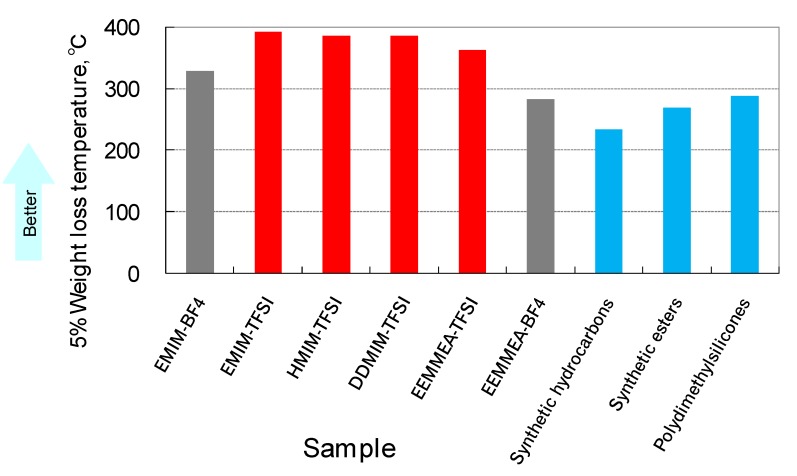
Thermogravimetric analysis: Temperature for 5% weight loss.

When solid surfaces rub against each other, friction heat is generated. This causes changes in the viscosity of the lubricant. Furthermore, auto-oxidation of the lubricant is promoted at elevated temperatures. These changes have an adverse effect on the lubrication properties. Therefore, thermo-oxidative stability of the lubricant is one of the important properties for practical lubricants. Ionic liquids have greater thermal stability than conventional synthetic lubricants, as shown in Figure 8. Interestingly, the stability of an ionic liquid depends on the structure of both the anion and the cation. The stability of TFSA is considerably higher than that of BF_4_. On the other hand, 1,3-dialkyl-imidazolium is more stable than quaternary ammonium. Although thermal analyses are convenient, evaluation by scale-up procedures is necessary for practical applications.

**Figure 9:**
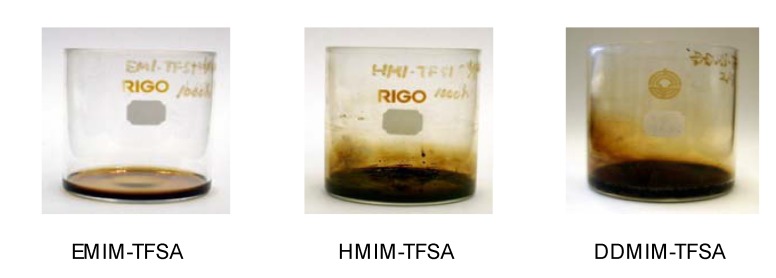
Photograph of samples kept at 200 °C for 1,000 h.

**Figure 10:**
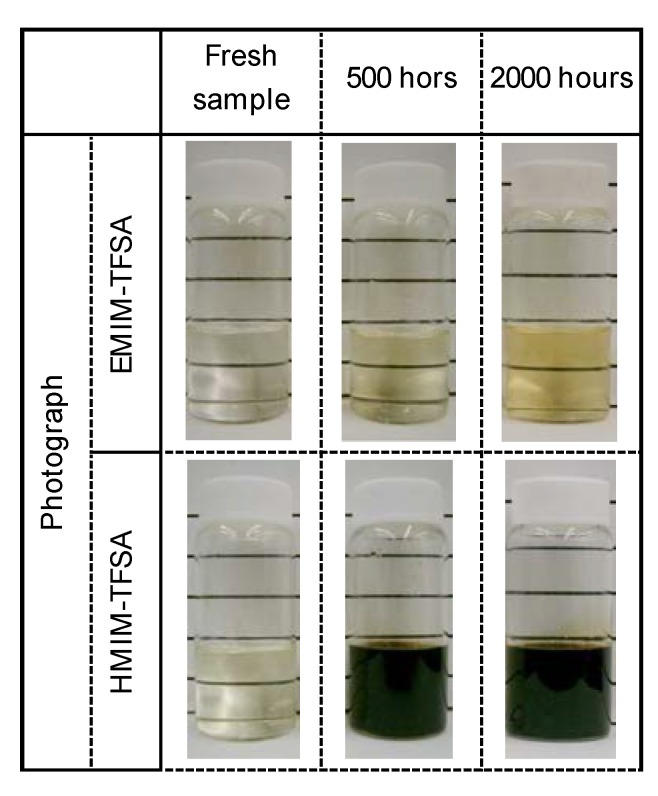
Photographs of samples kept at 150 °C.

**Figure 11:**
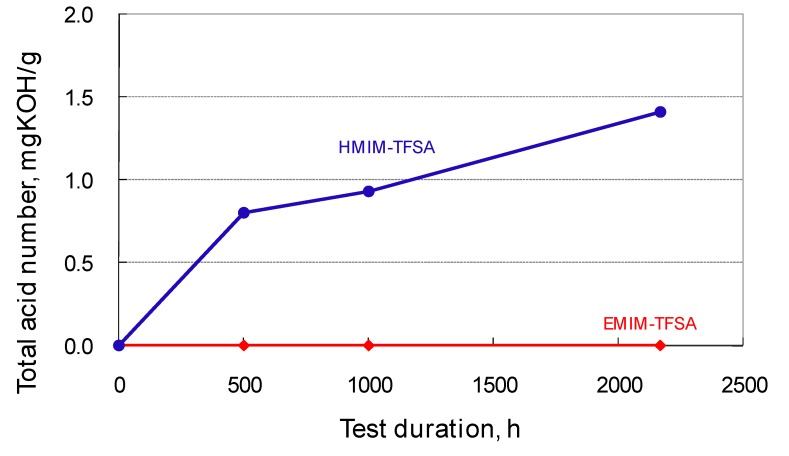
TAN trace during thermal stress test at 150 °C.

Figure 9 shows the resultant sample heated at 200 °C for 1,000 h in air. [EMIM]TFSA transformed into a dark liquid, while [HMIM]TFSA and [DDMIM]TFSA formed a solid deposit during the test. The same tendency was observed even at low temperatures. As shown in Figure 10, [HMIM]TFSA turned brown within 500 h of the test duration, while [EMIM]TFSA was clean even after 2,000 h. An increase in TAN was detected during oxidation of [HMIM]TFSA (Figure 11). 1-Methylimidazole was found in the thermal stressed [HMIM]TFSA at 150 °C. The scission of the C-N bond suggests the decomposition of imidazolium cation, which was initiated at the alkyl chain [14].

**Figure 12:**
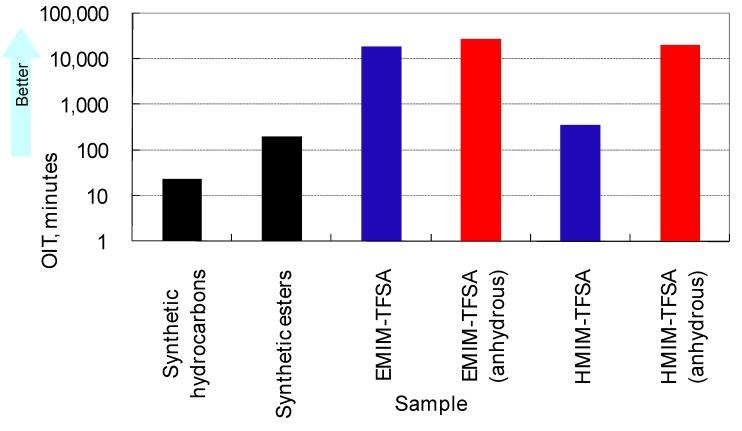
Results of the Rotating Bomb Oxidation Test.

The rotating bomb oxidation test regulated by the ASTM D2272 standard, which is one of the standard procedures used for evaluating the thermo-oxidation stability of practical lubricants [77], was employed. 50 g of the sample, 5 mL of water, and 52 g of copper wire were placed in an autoclave made of stainless steel. Oxygen was introduced at 620 kPa, which was set as the initial pressure. The autoclave was rotated at 150 °C and the oxygen pressure inside the reactor was monitored. The test was continued until the pressure dropped to 453 kPa. The test duration was reported as the oxidation induction time (OIT). As shown in Figure 12, [EMIM]TFSA is more stable than the conventional synthetic lubricants (y-axis is in log scale). In this test, [HMIM]TFSA is less stable than [EMIM]TFSA, especially in the presence of water. The results clearly indicate that water has a negative influence on the stability of ionic liquids. This might be one reason why hydrophobic anions are superior to hydrophilic ones in terms of stability.

## Tribochemical reactions of ionic liquids

**Figure 13:**
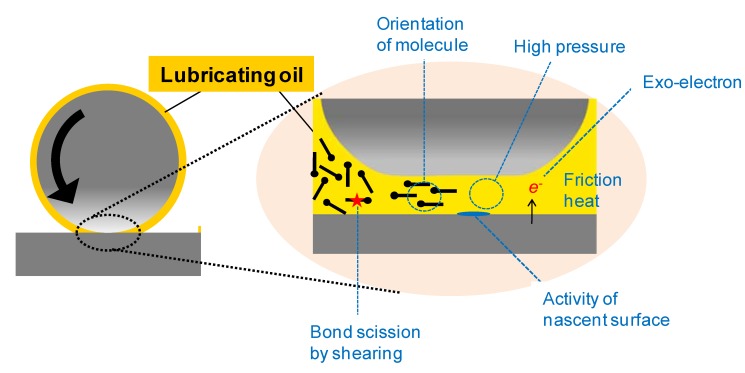
Model for rubbing surface as reaction zone.

A tribochemical reaction is a chemical reaction of lubricants that takes place on rubbing surfaces. Therefore, lubricants are called as reactants and the rubbing surfaces are considered to be the reactor. In a tribological reaction, mechanical energy and its derivatives (induced by friction) provide activation energy for the reaction [78]. The heat generated due to friction is one of the plausible sources of energy that provide activation energy for chemical reactions. Lubricating oil that is applied between rubbing surfaces is exposed to extreme pressures of up to several gigapascal. Certain high pressure reactions may take place under these conditions. Emission of exoelectrons caused by mechanical stress to crystalline solids is known. This leads to electrochemical reaction of lubricants. Direct scission of the C-C bonds in a polymer due to shear stress is also known. Orientation of molecules is possible when they are applied between surfaces. When this happens, selective or specific reactions take place. In practice, these properties were discovered individually, as schematically shown in Figure 13. Seemingly, these phenomena are complex and chaotic. However, lubricant chemists empirically make use of the tribochemical reactions. For example, certain antiwear films are produced by tribochemical reactions of anti-wear additives under tribological conditions. This corresponds to an in situ chemical modification of the surface through the tribochemical reaction of the additive.

The yield and selectivity of a tribochemical reaction are usually too low to allow isolation of any product(s), so reactions are traced by surface analysis instead of isolating the product(s). Among the several sophisticated instrumental analyses found in literatures, X-ray photoelectron spectroscopy (XPS) is one of the most frequently used technique applied in tribochemistry. By using this technique, we can study the chemical state of elements on the nanometer scale. Time of flight secondary ion mass spectroscopy (TOF-SIMS) is a high-resolution mass spectroscopy method used for analysis of uppermost surfaces with a depth of 1 nm. This method can be used to identify organic compounds on the rubbed surface if the fragmentation of the molecule is well studied.

As mentioned above, ionic liquids are stable compounds.; however, individual researchers have reported tribochemical reactions of ionic liquids [5,6,8,19]. Reactions of anionic moieties have been reported in most literatures, while reactions of cationic moieties were also observed [41].

**Figure 14:**
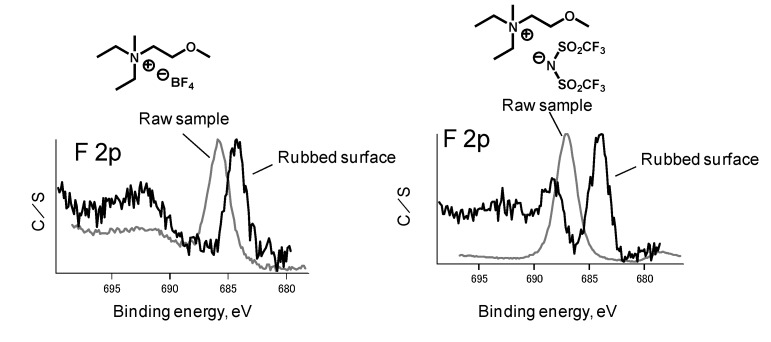
XPS spectra of rubbed surface.

Figure 14 shows the XPS spectra of a rubbed steel surface lubricated with BF_4_ salt and TFSA salt [15]. A considerable amount of fluorine was detected on the rubbed surfaces. “Raw” represents the reference ionic liquid applied on the steel surface before rubbing. The binding energy at 686 eV and 688 eV correspond to the fluorine in BF_4_ and TFSA, respectively. The spectra clearly show a change in the chemical structure of fluorine after rubbing. Both anions transformed to metal fluoride (binding energy at 684 eV) through a tribochemical reaction. It should be noted that the organic fluoride at a binding energy of 689 eV remains on the rubbed surface lubricated with TFSA salt. However, strong evidence of the tribochemical reaction of the cationic moiety could not be found under these conditions.

**Figure 15:**
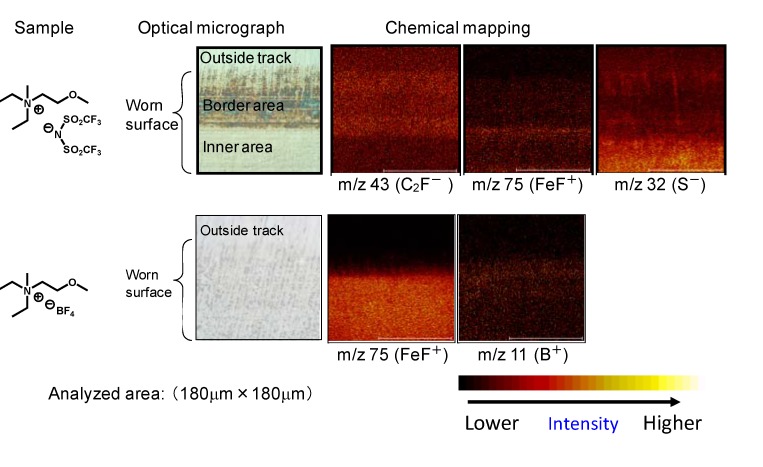
Chemical mapping of rubbed surface by TOF-SIMS.

TOF-SIMS analysis can be used to study the chemical contents on the rubbed surface in detail. Figure 15 shows chemical mapping of the rubbed surface lubricated with BF_4_ salt and TFSA salt. FeF^+^ at m/z 75 was found on both surfaces lubricated with BF_4_ salt and TFSA salt. B^+^ at m/z 11 was also found on the surface lubricated with BF_4_ salt. These fragments were not found on the outside wear track. These results strongly suggest that tribochemical reaction of the anionic moiety occurred. A detailed study of the chemical mapping revealed the differences in the chemical species between the inner and border areas of the rubbed surfaces lubricated by TFSA salt. Note that the inner area is exposed to a high load, and hence the friction conditions are severe. The intensities of FeF^+^ and S^-^ in the inner area were higher than the intensities in the border area. These results suggest that tribochemical reaction of TFSA took place in this area. On the other hand, a higher amount of organic fluoride was detected in the border area. Adsorption of TFSA appeared to take place preferentially in this area. The formation of FeF_2_ on the rubbed surface may help to improve tribological properties since the salt acts as a protective film. However, the salt is a Lewis acid that causes degradation of the lubricant and causes corrosion of the tribomaterial [19].

## Conclusions and Prospects

**Figure 16:**
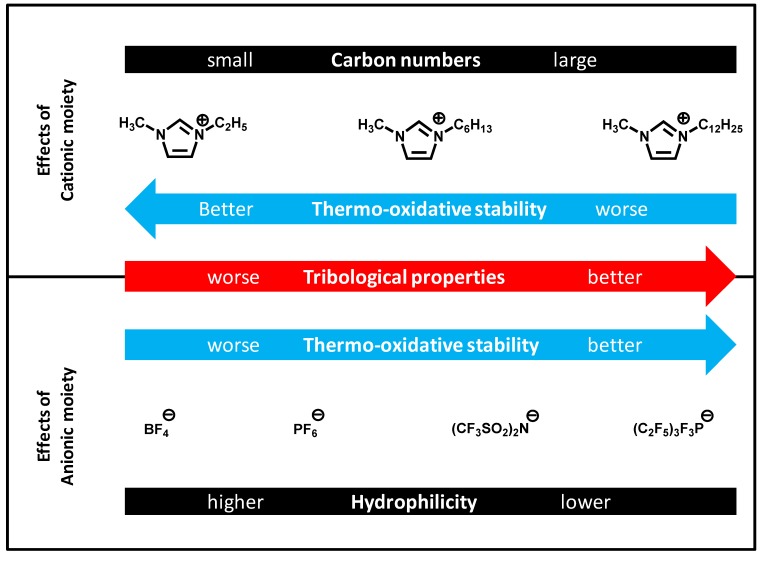
The relation between structure and lubricant properties.

The use of ionic liquids in tribology has been steadily increasing. Their properties as lubricants have been evaluated in laboratories. Figure 16 shows the relationship between the molecular structure of ionic liquids and their performance. Ideally, the anionic moieties should be hydrophobic to improve the tribological properties and the thermo-oxidative stability. A higher alkyl group in imidazolium improves the tribological properties, while it causes a decline in thermo-oxidative stability. Anti-wear properties of ionic liquids can be improved by means of additive technology. There is still room for optimization especially in the area of friction reduction and corrosion inhibition.

Since ionic liquids are recognized as designer fluids, considerable efforts need to be made to develop novel lubricants. For example, halogen-free anions are highly desired. Cationic moieties are more flexible than anionic moieties, and complex cations containing a multi-functional group are of interest to researchers.

Ionic liquids can be used as lubricants in vacuum machines in space applications. They can also be used in high-temperature applications where fire risk is significant. Lubricants with extremely low vapor pressure are desirable for machines in clean rooms where hazard gasses are highly restricted.
